# Sanctions and sovereignty: Venezuela’s human health crisis

**DOI:** 10.1097/MS9.0000000000004922

**Published:** 2026-04-06

**Authors:** Aneeq Mahmood Khan, Ayesha Mustafa, Mahnoor Fatima

**Affiliations:** a Department of Medicine, Sargodha Medical College, Sargodha, Pakistan; b Department of Medicine, Fatima Jinnah Medical and Dental University, Lahore, Pakistan; c Department of Medicine, Shaheed Ziaur Rahman Medical College, Rajshahi Medical University, Bogura, Bangladesh

Unilateral economic sanctions imposed on Venezuela appear to have contributed profound adverse effects on the health and human rights of its population, extending far beyond their intended political objectives and undermining the right to health enshrined in international law. While many sanctions regimes purport to include humanitarian exemptions for medical and food supplies, in practice, non-compliance by financial institutions and suppliers continues to create significant barriers to accessing essential health resources^[^1^]^. These impacts have intensified amid recent geopolitical developments, including the January 2026 U.S. operation that resulted in the capture of President Nicolás Maduro and other senior officials, raising complex legal and humanitarian concerns.

Recent analyses show that unilateral sanctions correlate with increased age-specific mortality and deterioration of public health systems, with annual excess deaths comparable to those seen in armed conflicts, highlighting the severe health consequences of sanctions when protective measures are inadequate^[^2^]^.

Independent assessments by United Nations experts report that sanctions have caused critical shortages of medicines, surgical supplies, vaccines, and diagnostic reagents. Blocked transactions disrupted blood reagent procurement and delayed antibiotic, anesthetic, and tuberculosis treatments, placing hundreds of thousands at risk, while up to 2.6 million children missed routine immunizations against measles, rotavirus, and yellow fever^[^3^]^.

Health service disruptions in Venezuela occur amid a deteriorating public health context, where the 2026 UNICEF humanitarian appeal reports a maternal mortality ratio of approximately 227 per 100 000 live births, an under-five mortality rate around 22 per 1000 live births, and routine immunization coverage below 72%, all of which increase vulnerability to preventable disease and undermine fundamental human rights including the rights to life and health. While sanctions have exacerbated these challenges, the health crisis also stems from multiple contributing factors, including long-term government economic policies, mismanagement, corruption, preexisting economic instability, and the sharp decline in global oil prices, which collectively strained public health infrastructure and resources prior to and alongside the intensification of sanctions^[^4^]^.

Despite formal humanitarian exemptions, sanctions frequently fail to protect medical supply chains. Financial institutions and global suppliers often refrain from engaging with sanctioned countries out of fear of punitive enforcement, effectively nullifying exemptions and impeding the timely delivery of health commodities, a phenomenon documented across multiple sanctions regimes and confirmed in narrative literature reviews of sanctions’ health impacts^[^5^]^.

In view of these findings, there is an urgent imperative for policymakers and global health stakeholders to critically reassess the formulation and execution of unilateral sanctions. Robust, enforceable safeguards must be instituted to ensure uninterrupted access to essential medicines, vaccines, diagnostics, and medical technologies, alongside systematic, real-time surveillance of population health outcomes to prevent avoidable morbidity and mortality. Such protective mechanisms are essential for safeguarding fundamental human rights and for shielding vulnerable populations from preventable harm in contexts shaped by coercive foreign policy interventions. This letter is in line with the TITAN Guidelines on the need for transparency in AI use in health care^[^6^]^.

## Data Availability

Not applicable to this study.

## References

[1] OHCHR. A/HRC/59/58: situation of human rights in the bolivarian republic of venezuela - report of the united nations high commissioner for human rights (Advance unedited version). 2026 Jan 16. https://www.ohchr.org/en/documents/country-reports/ahrc5958-situation-human-rights-bolivarian-republic-venezuela-report

[2] Rodríguez F Rendón S Weisbrot M. Effects of international sanctions on age-specific mortality: a cross-national panel data analysis. Lancet Global Health 2025;13:e1358–66. 40712610 10.1016/S2214-109X(25)00189-5

[3] Progressive International. UN expert releases full report on impact of US-led sanctions against venezuela. 2026 Jan 16. https://progressive.international/wire/2021-10-18-un-expert-releases-full-report-on-impact-of-us-led-sanctions-against-venezuela/en/

[4] Bolivarian Republic of Venezuela Appeal | UNICEF. 2026 Jan 16. https://www.unicef.org/appeals/venezuela#download

[5] Yazdi-Feyzabadi V Zolfagharnasab A Naghavi S. Direct and indirect effects of economic sanctions on health: a systematic narrative literature review. BMC Public Health 2024;24:2242. 39154171 10.1186/s12889-024-19750-w PMC11330615

[6] Agha RA Mathew G Rashid R. Transparency In The reporting of Artificial INtelligence – the TITAN guideline. Prem J Sci 2025;10:100082.

